# Actinomycosis: Mimicking Malignancies in Multiple Anatomical Sites—A Three-Patient Case Series

**DOI:** 10.3390/medicina61020256

**Published:** 2025-02-02

**Authors:** John Fernando Montenegro, Vanessa Correa Forero, Yamil Liscano, Andres Grueso Pineda, Diana Marcela Bonilla Bonilla, Paola Andrea Ruiz Jimenez

**Affiliations:** 1 Specialization in Internal Medicine, Department of Health, Universidad Santiago de Cali, Cali 5183000, Colombia; investigacion@clinicadeoccidente.com (V.C.F.); andresgruesopineda@gmail.com (A.G.P.); diana.bonilla01@usc.edu.co (D.M.B.B.); parj85@gmail.com (P.A.R.J.); 2 Department of Research and Education, Clínica de Occidente S.A., Cali 760046, Colombia; 3 Genetics, Physiology, and Metabolism Research Group (GEFIME), Ciencias de la Salud Universidad Santiago de Cali, Cali 5183000, Colombia; 4 Grupo de Investigación en Salud Integral (GISI), Departamento Facultad de Salud, Universidad Santiago de Cali, Cali 5183000, Colombia

**Keywords:** actinomyces, contagion, malignancy, neoplasia, granulomatosis, pathology

## Abstract

*Background and Objectives:* Actinomycosis is a rare chronic contagion caused by *Actinomyces* spp. known for its ability to mimic malignant processes across various anatomical locations. Its clinical presentation can often resemble malignancies, *Mycobacterium tuberculosis* infections, nocardiosis, fungal infections, or other granulomatous diseases. This case series presents three patients diagnosed with *Actinomyces* spp., highlighting the diagnostic challenges and diverse clinical manifestations of the disease. *Materials and Methods:* We reviewed the clinical course, diagnostic procedures, and treatment outcomes of three patients with confirmed *Actinomyces* spp. The first case involved a 51-year-old male with a history of rhabdomyosarcoma in remission who presented with dysphagia. Magnetic resonance imaging identified an irregularly enhancing mass in the tonsil, and subsequent tonsillectomy confirmed *Actinomyces* spp. The second patient, an 80-year-old female, presented with dysphagia and a sublingual mass initially suspected to be diffuse large B-cell non-Hodgkin lymphoma; however, a histopathological analysis confirmed *Actinomyces* spp. The third case involved a 72-year-old male with abdominal pain and an ulcerated gastric lesion, where subtotal gastrectomy and histopathological examination confirmed the diagnosis of *Actinomyces* spp. *Results:* These three cases highlight the ability of *Actinomyces* spp. to closely mimic malignant lesions, which significantly complicates the diagnostic process. Although personalized interventions were required for each patient, diagnoses were ultimately confirmed through histopathology. Despite these challenges, timely recognition and appropriate treatment were achieved, underscoring the need to consider *Actinomyces* spp. in the differential diagnosis of similar presentations. *Conclusions:* *Actinomyces* spp. remains a diagnostic challenge due to its ability to mimic a variety of malignant and contagion conditions. This case series emphasizes the need for a thorough histopathological examination and a high index of suspicion when encountering lesions with atypical presentations. Given the potential for misdiagnosis, awareness and consideration of *Actinomyces* spp. are crucial in the differential diagnosis of chronic contagion and mass lesions. Further studies are warranted to refine diagnostic and therapeutic approaches.

## 1. Introduction

*Actinomyces* spp. is an uncommon, slowly progressing contagion characterized by chronic suppurative lesions [1]. It is caused by anaerobic or microaerophilic bacteria, primarily of the genus Actinomyces, which belong to the family *Actinomycetaceae* and are recognized as Gram-positive filamentous anaerobes [2]. This infection is notable for its ability to mimic malignant processes, leading to significant diagnostic challenges.

The condition is more prevalent in individuals with poor oral hygiene, those with a history of dental procedures, or those with underlying conditions that compromise the integrity of mucosal barriers, such as diabetes mellitus or immunosuppression. Additionally, chronic use of intrauterine devices has been linked to pelvic actinomycosis in women [3,4,5]. The contagion is most commonly found in rural areas, where individuals are more likely to have direct contact with soil and animals, both of which can harbor *Actinomyces* spp. Epidemiologically, actinomycosis has a higher incidence in men, particularly those between the ages of 40 and 60, possibly due to occupational exposures and behaviors that increase the likelihood of mucosal injury or contamination [6]. The disease has the potential to mimic other conditions, such as malignant neoplasms, active contagion with *Mycobacterium tuberculosis,* non-tuberculous mycobacteria, nocardiosis, fungal infections, or other granulomatous diseases [7,8].

The clinical presentation of *Actinomyces* spp. is particularly challenging due to its variability and the involvement of different anatomical sites. The clinical manifestations depend on the location of the contagion and may be accompanied by general symptoms such as asthenia, adynamia, weight loss, febrile peaks, and chronic suppurative granulomatous masses [2,9].

Diagnosing *Actinomyces* spp. in a timely manner is often difficult, leading to delays that can extend for months. This delay is primarily due to the nonspecific nature of the clinical manifestations. Additionally, culture techniques are infrequently used because Actinomyces is a bacterium with slow growth, resulting in a poor diagnostic yield. Although diagnostic imaging can guide the diagnosis, the findings are often indeterminate. The gold standard for diagnosis remains histopathology, utilizing stains such as Grocott, Gomori, and hematoxylin–eosin, which can reveal Actinomyces colonies as radiating structures or “sunburst” patterns with fine, branching filaments; sulfur granules; and chronic inflammatory reactions [2,10,11].

Treatment is based on the prolonged use of beta-lactam antibiotics, with penicillin G being the treatment of choice in most cases. Alternatives include third-generation cephalosporins and carbapenems; however, the latter should be used with caution due to the growing issue of bacterial resistance. Initial management typically involves intravenous administration for 4 to 6 weeks, followed by oral therapy to complete a total treatment duration of 6 to 12 months. In some instances, antibiotic therapy may be complemented with surgical intervention depending on the severity and extent of the disease [8,12].

The primary objective of this case series is to illustrate the various clinical presentations of *Actinomyces* spp., a contagion entity that, despite being curable, poses significant diagnostic challenges. Familiarity with the diverse manifestations of *Actinomyces* spp. could facilitate earlier recognition and prevent complications, such as unnecessary surgeries, which occurred in two of the three cases presented in this report. By highlighting these cases, we aim to raise awareness of the diagnostic challenges and improve patient outcomes by promoting timely and accurate diagnosis.

## 2. Materials and Methods

This study was conducted in accordance with the Declaration of Helsinki (revised in 2013) and was approved by the ethics committee of Clínica de Occidente in Cali, Valle del Cauca, Colombia. Written informed consent was obtained from all patients. The case series includes patients diagnosed with *Actinomyces* spp. between 2022 and 2023, confirmed through final biopsy results. Clinical data collected for each patient included age, sex, underlying conditions, dental issues, history of trauma, previous contagion, infection location, symptoms, and symptom duration. Each patient underwent different examinations; for example, in case 1, blood cultures were used, which were negative, and finally histopathology. Another important aspect was the use of imaging; in case 1, MRI of the face and neck with contrast as well as PET-CT were used. Similarly, in case 2, PET-CT was used, and the results were finally confirmed with histopathology. In case 3, endoscopic studies, such as endoscopy and entero-MRI, were used, and the results were finally confirmed with gastric biopsy and histopathology. This case series was conducted using the CARE checklist.

## 3. Results

### 3.1. Cases

#### 3.1.1. Case 1

**Figure 1:**
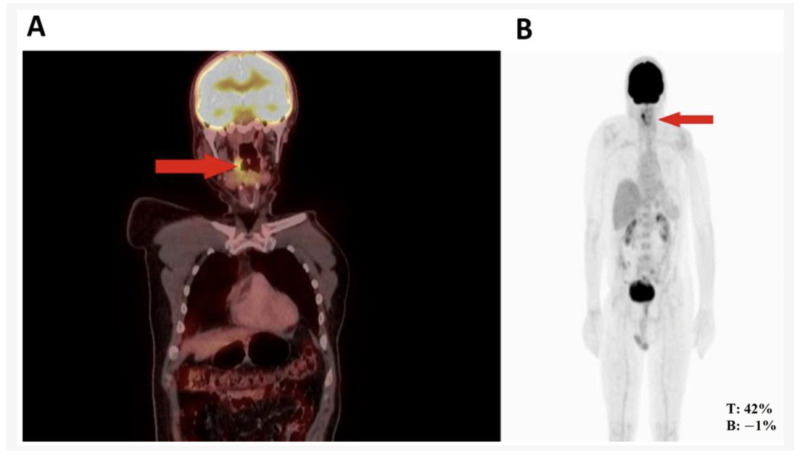
(**A**) Nodule in right pharynx and palatoglossal arch measuring 22 × 10 mm, SUV 6.5. (**B**) Hypermetabolic nodular thickening of right lateral pharyngeal wall, nonspecific, suspicious for neoplastic involvement. Taken from Montenegro et al. 2024 [13]. Used with permission.

The first case involves a 51-year-old man with a history of mandibular rhabdomyosarcoma and metastases to the left shoulder and right hip. Twenty-five years ago, he underwent a parotidectomy due to rhabdomyosarcoma and received chemotherapy and radiotherapy as part of his oncological treatment. In 2022, he experienced increased pain in his left shoulder with an intensity of 8/10, along with pain in his right hip that limited his mobility. He also reported dysphagia for both liquids and solids, as well as difficulty initiating swallowing, leading to an initial evaluation by orthopedic oncology. Due to worsening pain, a biopsy of the left proximal humerus was performed in June 2022. The immunohistochemistry analysis revealed a sparse presence of small blue tumor cells, with inconclusive results for sarcoma, prompting the request for a PET scan (Figure 1). The scan showed hypermetabolic nodular thickening in the right lateral pharyngeal wall, suspicious for neoplasia.

**Figure 2:**
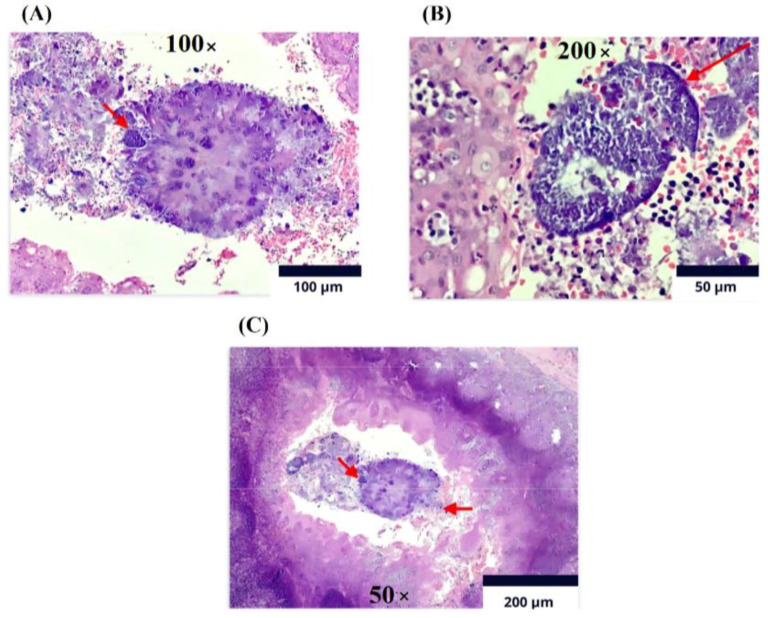
Immunohistochemistry (viewed using an Olympus CX33 microscope - Manufacturer: Olympus Corporation- City: Tokyo- Country: Japan ). Lymphoid tissue displaying follicles of varying sizes and shapes within cortex. (**A**) Magnified 100×: reactive germinal centers exhibiting significant phagocytosis and filamentous bacteria associated with *Actinomyces* spp. on surface of sulfur granules (red arrow). (**B**) Magnified 200×: germinal centers encircled by mantle of mature lymphocytes embedded within sulfur granules due to *Actinomyces* spp. (red arrow). (**C**) Magnified 50×: crypts containing debris, clusters of polymorphonuclear cells, and colonies of filamentous bacteria, consistent with Actinomyces spp. Left arrow points to polymorphonuclear reaction, while right arrow indicates presence of bacterial colonies. Taken from Montenegro et al. 2024 [13]. Used with permission.

Following these findings, an MRI of the paranasal sinuses was conducted in June 2022, revealing an irregularly enhancing mass affecting the buccopharyngeal space and the right palatine tonsil, extending to the base of the tongue. A right tonsillectomy was performed, and a biopsy with immunohistochemistry was requested to obtain a more accurate diagnosis. Histopathological analysis (Figure 2) of the palatine tonsil showed reactive follicular hyperplasia with the presence of filamentous bacterial colonies consistent with *Actinomyces*. Based on these results, the infectious disease service evaluated the patient and referred him to the emergency department to initiate induction antibiotic therapy with 2 g of ceftriaxone every 24 h for four weeks, administered via a peripheral venous catheter. The patient completed the induction phase without complications and continued with 2 g of oral amoxicillin every 12 h. He is currently in his eighth month of treatment and showing adequate clinical progression with normal renal and hepatic function tests. Subsequently, the patient resolved dysphagia and did not present febrile peaks.

#### 3.1.2. Case 2

**Figure 3:**
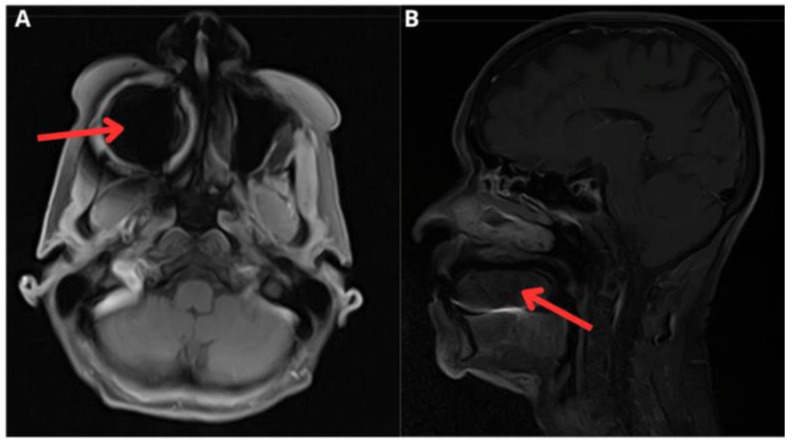
Contrast-enhanced MRI of the face. (**A**) Axial section. An image of the base of the tongue towards the right side, displacing the muscular structure of the tongue (red arrow). (**B**) Sagittal section. An ovoid-shaped image with well-defined borders, measuring 41 × 49 × 21 mm, corresponding to a presumably cystic lesion (red arrow).

An 80-year-old Caucasian woman living in an urban area, with a history of breast cancer resected 20 years ago and a right hip replacement, presented with a progressively enlarging sublingual ulcerated mass over the course of three months. The mass caused intense pain and dysphagia to solids and partially to liquids. Additionally, she experienced weight loss, decreased appetite, and dysphonia. She sought care in the emergency department, where she was evaluated by the maxillofacial surgery team. A facial MRI (Figure 3) revealed a cystic-appearing sublingual mass measuring 41 × 49 × 21 mm. Subsequently, a contrast-enhanced neck CT scan was performed, which characterized the lesion as a well-defined lobulated mass measuring 35 × 21 mm on the axial plane, with a 3 mm dilation of the submandibular duct. Extension studies, including a contrast-enhanced chest CT, ruled out further spread of the mass. In this context, an incisional biopsy of the lesion was performed, revealing neoplastic lymphoid cells with characteristics typical of diffuse large B-cell non-Hodgkin lymphoma of germinal center origin. Following sample study protocols, cultures and histopathological analysis confirmed the presence of a superinfection by *Actinomyces* spp. Hematology recommended specific oncological treatment with chemotherapy, while the contagion disease team prescribed induction antibiotic therapy with ceftriaxone for four weeks, followed by amoxicillin for six months.

After completing antibiotic treatment, the patient showed improvement in dysphagia for solids and in speaking without febrile peaks while receiving treatment. During nutrition via nasojejunal tube, she experienced diarrhea, which resolved, and after completing the antibiotic treatment, the nasojejunal tube was removed.

#### 3.1.3. Case 3

**Figure 4:**
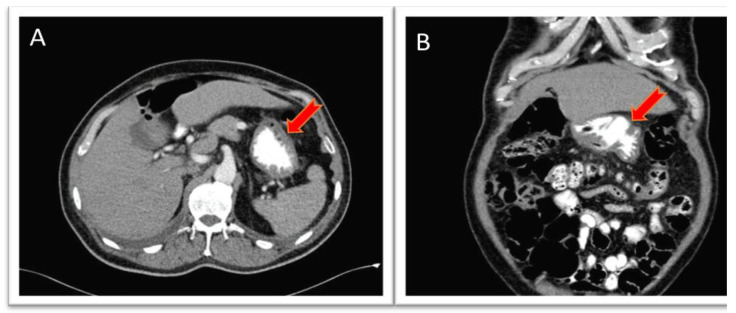
Abdominal computed tomography. (**A**) The axial view shows a poorly distended gastric chamber; however, a slight thickening of the gastric antrum walls is observed without a defined mass (red arrow). (**B**) The coronal view also reveals thickening of the gastric antrum walls (red arrow).

**Figure 5:**
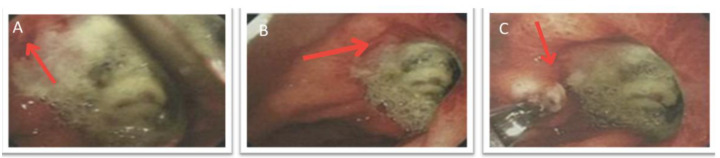
Esophagogastroduodenoscopy of the stomach. (**A**,**B**) The antrum exhibits patchy edema and erythema, and the stomach shows an extensive transmural ulcer surrounded by areas of fibrosis, neovascularization, and vascular congestion (red arrow). (**C**) A deep ulceration (3 × 4 cm) is present on the posterior surface of the distal antrum, with indurated borders, a necrotic base, and fibrin (red arrow).

A 72-year-old male patient with a history of chronic obstructive pulmonary disease (COPD) due to heavy smoking, as well as arterial hypertension, presented with a two-month history of intermittent abdominal pain associated with unintentional weight loss. His symptoms worsened, with increased pain accompanied by oral intolerance and episodes of hematemesis and melena, leading him to seek emergency care. Supportive measures were initiated for stabilization, and an esophagogastroduodenoscopy was performed, which documented a Forrest III antral ulcer. Pathological results reported non-erosive antral gastritis with negative *Helicobacter pylori*. Additionally, a CT scan of the abdomen revealed thickening of the gastric antrum walls (Figure 4). Based on these findings, the gastroenterology service decided to initiate medical management with proton pump inhibitors, which resulted in partial pain relief; however, episodes of melena persisted. Consequently, a repeat upper endoscopy was considered, revealing a more extensive ulcerative lesion suggestive of malignancy (Figure 5), with pathology findings indicating chronic granulomatous inflammation without evidence of neoplasia.

**Figure 6:**
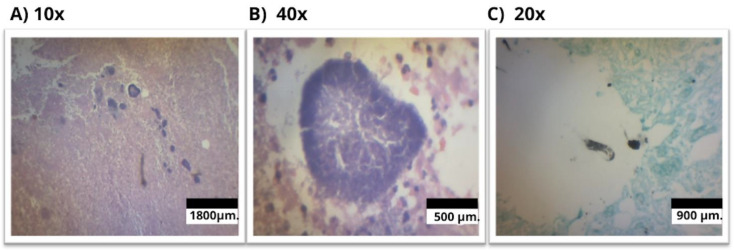
Immunohistochemistry of lymphoid tissue displaying follicles of various sizes and shapes within the cortex. (**A**) Magnification at 10× with hematoxylin-eosin. The ulcer bed shows reactive germinal centers with marked phagocytosis and filamentous bacteria consistent with *Actinomyces* on the surface of sulfur granules (the red arrow indicates the bacterial colony). (**B**) Magnification at 40× with hematoxylin–eosin. The germinal centers are encased by a mantle of mature lymphocytes, surrounded by sulfur granules, along with a lymphoplasmacytic infiltrate accompanied by abundant epithelioid histiocytes and occasional foreign body-type multinucleated giant cells. A small focus with *Actinomyces* is identified due to the infection (the red arrow indicates the bacterial colony). (**C**) Magnification at 20×. The crypts reveal debris, clusters of polymorphonuclear cells, and colonies of filamentous bacteria compatible with *Actinomyces* spp., highlighted by methenamine silver staining (the red arrow indicates the bacterial colony).

However, as progress remained unsatisfactory despite medical treatment and as the gastric ulcer continued to grow, a third upper endoscopy was performed by the gastric surgery service, revealing further ulcer extension with necrotic areas. Given this evolution, the lack of a confirmed diagnosis, the persistence of symptoms, and the rapid progression of the gastric ulcer, the case was presented at an interdisciplinary medical board meeting. It was decided to proceed with a subtotal gastrectomy, during which a histopathological examination confirmed a diagnosis of *Actinomyces* spp. (Figure 6). Based on these findings, the infectious disease team recommended antibiotic therapy with intravenous ceftriaxone at a dose of 2 g for six weeks, followed by 2 g of oral amoxicillin every 12 h for a total treatment duration of one year. The patient’s clinical course improved, leading to the complete resolution of symptoms. Subsequently, after completing the antibiotic treatment, the patient was evaluated by the infectious disease service with an adequate clinical progression, with no fever, no pain, no gastrointestinal symptoms, weight gain, and laboratory tests showing a normal blood count, normal liver and kidney function, and negative CRP. Previous reports included an abdominal CT scan with post-surgical changes from gastrectomy and a new endoscopy showing post-surgical changes, with endoscopic pathology showing no signs of aplasia, leading to the conclusion that the infection was treated.

The analysis of three *Actinomyces* spp. cases revealed both commonalities and significant differences, highlighting the complexity of this contagion (see Table 1). In all instances, the diagnosis was confirmed histopathologically, and a similar antibiotic regimen, starting with ceftriaxone and followed by amoxicillin, was effective, leading to symptom improvement in all patients. Despite this, the initial symptoms and affected organs varied: the first case involved the tonsil, the second the base of the tongue, and the third the jejunum, an atypical location for *Actinomyces* spp. The third case’s unusual site and the first patient’s history of rhabdomyosarcoma, which initially suggested malignancy, underscore the diagnostic challenges posed by *Actinomyces* spp., particularly when it mimics neoplastic conditions or occurs in uncommon locations.

## 4. Discussion

The three cases presented in this series highlight the diagnostic and therapeutic challenges associated with actinomycosis, particularly when it mimics malignant processes in patients with complex medical histories. In the first case, a middle-aged man with a history of cancer exhibited symptoms initially suggestive of neoplastic recurrence. However, the definitive diagnosis of Actinomyces spp. was established through histopathology, emphasizing the importance of considering bacterial infections in such contexts (Samaila et al., 2023 [14]; Mohanty, 2006 [15]). This case underscores the necessity for clinicians to maintain a broad differential diagnosis, even in patients with prior malignancies.

Similarly, the second case involved an elderly woman with a sublingual mass suspected to be diffuse large B-cell non-Hodgkin lymphoma. The concomitant presence of Actinomyces spp. required a complex therapeutic approach combining chemotherapy and antibiotics, aligning with findings from Puri et al. (2015) [16] and Bajpai et al. (2023) [17]. The coexistence of a non-Hodgkin lymphoma added complexity to the therapeutic management, underscoring the need for integrated treatment strategies in managing dual pathologies.

In the third case, a 72-year-old man with nonspecific gastrointestinal symptoms was postoperatively diagnosed with actinomycosis. This scenario highlights the difficulty in distinguishing this infection from malignant neoplasms in gastrointestinal presentations. Similar challenges have been noted in studies by Al-Obaidy et al., 2015 [18]; Chernopolsky et al., 2021 [19]; and Eskarous et al., 2021 [20], emphasizing that actinomycosis can mimic malignancies in the digestive tract and complicate the diagnostic process.

The risk factors for actinomycosis include poor dental hygiene, oral surgery, and the use of intrauterine devices [21,22]. Diagnosing actinomycosis can be challenging due to its nonspecific clinical manifestations and its ability to imitate malignancies. The presence of sulfur granules is pathognomonic and aids in diagnosis [23]. However, imaging and endoscopic evaluations often fail to differentiate actinomycosis from tumors, necessitating histological confirmation. In our series, all diagnoses were made using hematoxylin-eosin staining, which is crucial since cultures can yield negative results in up to 76% of cases [24]. Advanced molecular techniques, such as 16S rRNA sequencing and MALDI-TOF mass spectrometry, have enhanced diagnostic accuracy and speed, providing precise identification at the genus level [25,26,27].

Regarding the management of systemic actinomycosis, current clinical guidelines recommend the prolonged use of beta-lactam antibiotics, with penicillin G as the preferred option and third-generation cephalosporins as alternatives in cases of allergy [8,11,28]. The treatment duration is typically extended, involving an intravenous induction phase of 4 to 6 weeks followed by oral therapy for 6 to 12 months to ensure complete eradication of the infection [22,29]. This approach was effective in all patients in our series, who initially received ceftriaxone, followed by amoxicillin, resulting in significant clinical improvement.

While the cervicofacial region is commonly affected by actinomycosis, other anatomical sites can also be involved [30]. In severe cases, surgical intervention may be necessary, particularly when there is extensive tissue involvement or inadequate response to antibiotic therapy [23]. Surgical debridement has been shown to reduce antibiotic therapy duration and improve wound healing [31]. Therefore, a combination of surgical debridement and intravenous antibiotics, followed by 3 to 6 months of oral antimicrobial agents, is widely recommended for effective management [32].

The clinical implications of these findings emphasize the importance of considering *Actinomyces* spp. in the differential diagnosis of chronic masses and ulcers, especially in patients with risk factors such as immunosuppression or a history of malignancy. Early identification and appropriate management can prevent unnecessary surgical interventions and improve patient quality of life, as supported by the existing literature [8,11,26]. Clinicians should maintain a high index of suspicion for underlying malignancies and ensure close clinical and radiological follow-ups to confirm complete infection resolution and rule out any hidden malignancies (Drozdowicz et al., 2021 [24,25]).

Additionally, Actinomyces is frequently associated with medication-related osteonecrosis of the jaw (MRONJ), with a prevalence of 82.18% reported in one study [33]. Early identification and appropriate management, typically involving prolonged antibiotic therapy, can improve patient outcomes and prevent unnecessary surgical procedures [20,34].

Recent advancements in molecular diagnostic techniques have significantly improved the detection and identification of infections like actinomycosis. Metagenomic next-generation sequencing has emerged as a rapid and effective tool for identifying pathogens in clinical samples, potentially reducing misdiagnosis and guiding targeted treatment [35]. These methods, along with other molecular techniques like targeted NGS, complement traditional diagnostic approaches and are particularly valuable in rare or difficult-to-diagnose infections [27]. By providing more accurate identification, advanced molecular methods are increasingly replacing traditional biochemical tests and improving patient management in suspected infectious diseases.

The limitations of this study include its retrospective nature and the small sample size of only three cases, which limit the generalizability of the results. Such a limited number of cases may not fully reflect the clinical variability of actinomycosis in the general population. Therefore, prospective studies with larger cohorts are needed to explore the full clinical spectrum of the disease and to optimize diagnostic and therapeutic strategies.

## 5. Conclusions

The findings from this case series emphasize the diverse clinical presentations and diagnostic complexities of *Actinomyces* spp. a rare contagion disease that can closely mimic malignancies. In all three cases, histopathological confirmation was crucial for diagnosis, as imaging and endoscopic findings alone were insufficient to differentiate *Actinomyces* spp. from tumors. The cases highlighted the effectiveness of prolonged antibiotic therapy with beta-lactams, which led to complete symptom resolution in all patients. These results align with the existing literature that highlights the importance of accurate biopsy-based diagnosis and appropriate antibiotic management in treating *Actinomyces* spp. Future research should focus on expanding case studies and exploring the use of molecular techniques for more rapid and precise diagnosis.

## Figures and Tables

**Table 1:** Comparative clinical overview of three *Actinomyces* spp. cases.

Characteristics/Case	Case #1	Case #2	Case #3
Age/gender	51 years/male	80 years/female	72 years/male
Occupation	Retired	Housewife	Merchant
Year of consultation and diagnosis	2023	2024	2021
Admission symptoms	Dysphagia and chronic parieto-occipital headache	Painful sublingual mass, progressive growth, dysphagia, hyporexia, and weight loss	Weight loss, abdominal pain, hematemesis, and melena
Diagnostic aids	MRI of face and neck with contrast showing irregular enhancement measuring approximately 25 × 25 × 16 mm in pharyngeal mucosal space involving right palatine tonsil and base of tongue	PET CT * showing hypermetabolic nodule of right pharynx and palatoglossal arch measuring 22 × 10 mm	Endoscopy: moderate, active, non-atrophic chronic gastritis; no malignant neoplasia; Entero-MRI: distended stomach with compact image inside, nonspecific thickening of jejunal loops; contrast-enhanced abdominal CT
Affected structures or organs	Tonsil	Base of tongue	Jejunum
Sample	Biopsy of tonsil	Biopsy of base of tongue	Biopsy of gastric tissue
Cultures—incubation time	Blood cultures, 5/5 negative—14 days of incubation	No	No
Histopathology diagnosis	*Actinomyces* spp., reactive follicular hyperplasia	*Actinomyces* spp.	*Actinomyces* spp.
Management	Induction: ceftriaxone for 4 weeks Consolidation: amoxicillin for 12 months	Induction: ceftriaxone for 4 weeks; consolidation: amoxicillin for 6 months	Induction: ceftriaxone for 6 weeks Consolidation: amoxicillin for 12 months
Outcome	Notable improvement in symptoms	Increased appetite, weight gain, regular bowel habits, no new episodes of hematemesis or melena	Notable improvement in symptoms
Final condition	Alive	Alive	Alive

* PET CT: positron emission tomography and computed tomography.

## Data Availability

All data are contained within the article.
